# Holistic Understanding of the Role of Carbohydrate Antigen 19-9 in Pancreatic Cancer Screening, Early Diagnosis, and Prognosis: A Systematic Review

**DOI:** 10.7759/cureus.44382

**Published:** 2023-08-30

**Authors:** Lakshmi Sai Niharika Janga, Hembashima G Sambe, Mohamed Yasir, Ruzhual K Man, Amaresh Gogikar, Ankita Nanda, Lubna Mohammed

**Affiliations:** 1 Research, California Institute of Behavioral Neurosciences & Psychology, Fairfield, USA

**Keywords:** pancreatic malignancy, tumor marker, role in early diagnosis, ductal pancreatic adenocarcinoma, pancreatic cancer prognosis, screening test, ca-19-9

## Abstract

Pancreatic ductal adenocarcinoma (PDAC) is a significant challenge due to its silent progression and well-advanced, unresectable, complicated presentation. Detecting this disease early on is crucial, and researchers have been investigating various potential biological markers, such as carbohydrate antigen 19-9 (CA 19-9), hoping to find indicators that can aid in its early detection. The primary focus of this review is on the diagnostic usefulness of CA 19-9 in detecting pancreatic cancer (PC) in the beginning stage and its usefulness in predicting progression. The database search of articles from PubMed, PMC, the Cochrane Library, and Google Scholar identified 227 articles published from 2013 to 2023. The keyword mix used in the search technique included terms like "CA 19-9," "pancreatic cancer," "diagnosis," and "early detection." This study provides evidence of CA 19-9's ability in detecting PDAC in the pre-diagnostic stage. But since the outcomes were inconsistent among the included trials, further analysis is required to develop standardized diagnostic criteria and methodologies. Furthermore, because of the variability of the study, it is not easy to make firm conclusions on CA 19-9's sensitivity as well as specificity in the first stage of pancreatic neoplasm. This in-depth overview of the available literature provides new insights into using CA 19-9 as a biological marker for detecting undiagnosed PC before progressing into the advanced stage, and was proven beneficial. However, this has to be shown in broader research with adequate sample size. Although it shows promise as a diagnostic tool, further study is required to confirm these findings.

## Introduction and background

When discussing the universal cancer death toll, pancreatic cancer is an imperative contributor. The estimated incidence was 495,773 newly reported instances in 2020. On the other hand, the deaths attributed to pancreatic cancer were 466,003 [1]. The illness is aggressive and lethal. It can be inferred by the data trends from the United States showing high fatalities (48,220) and virtually identical incidence (60,430), which suggests its rapid progression from diagnosis to death. Advanced pancreatic tumors have a five-year survival rate of around 10% [2]. The outlook for those suffering from pancreatic tumor is not good, which might be linked to several factors, including non-specific symptoms leading to delayed diagnosis at advanced stages, smoking, chronic alcoholism, and the absence of reliable biomarkers capable of detecting the disease in its precursor forms or early stages [3]. Pancreatic cancers may be detected using a wide variety of telltale markers. Most of the potential biomarkers lack extensive validation on a large scale. Only a small subset has been subjected to thorough research, garnering most of the study's focus.

Carbohydrate antigen 19-9

Carbohydrate antigen 19-9 (CA 19-9), well known as the sialo ganglioside, was initially discovered in 1979 by Koprowski et al. in the SW1116 colon cancer cell line while utilizing the hybridoma method to detect the molecule [4]. CA 19-9 is produced during the synthesis of its analog, disialyl Lewis a (di-sLea), which has an additional sialic acid structure attached. The usual lining of the gastrointestinal tract has di-sLea, which functions as a ligand for the immune cells. During the early stage of carcinogenesis, epigenetic silencing of the gene that translates into the enzyme 2-6 sialyltransferase inhibits the production of disialyl Lewis-a. Consequently, sialyl Lewis a (sLea), also known as CA 19-9, is produced abnormally and accumulates in the body. sLea also functions as a ligand for E-selectin, a protein found on endothelial cells of blood vessels that facilitates tumor cell adhesion. This mechanism proves its contribution to cancer invasion and metastasis [5]. Lewis (α- β+) and Lewis (α+ β-) are the blood groups that have Lewis blood group antigens and are associated closely with the CA 19-9 bioparameter. CA 19-9 has limited applicability in the population with the (α-β-) blood group as these individuals are deficient in enzyme 1,4 fucosyl-transferase, which is essential for antigen formation. Since this group makes up 10% of the population, universal recognition of CA 19-9's role as a diagnostic biomarker is restricted [6].

Physiology and pathophysiology of CA 19-9

CA 19-9, or sLea glycan antigen, can be synthesized as a glycolipid (with the ceramide component incorporated into lipid bilayers of cell membranes) or as a glycoprotein (most typically O-linked to mucins). To avoid interference with antibodies and their effector system, sLea expression in normal tissues is confined to the ductal epithelium of tissues. However, it is expressed in many different types of gastrointestinal cancers, thus serving as a serum indicator of cancer. sLea molecules attached to normal cells of epithelium act as epitopes for these recognition receptors of leukocytes (endothelial E-selectin) and further aid in immunoregulation. This property is lacking in glycans expressed by malignant cells [7]. CA 19-9 assays rely on the detection of a glycan, namely, the sialyl Lewis a, the antigen of interest in the CA 19-9 test. To improve upon the CA 19-9 test, Tang et al. proposed identifying prominent biomarkers in patients with low sLea (∼25% of cancer cases). Tumors deficient in sLea produce glycans that are structurally similar to sLea but cannot be picked up by CA 19-9 antibodies. Motif profiling was used to determine the structural isomer of sLea, sialyl Lewis x (sLex). It was found to be overrepresented in the plasma of some sLea-deficient cancers in up to 14%-19% of patients [8].

It is well known that during malignant transformation, cell surface glycans experience considerable changes. This glycosylation alteration is a hallmark of the transition to malignancy. Malignant cells alter their glycans in a number of ways, most often by modifying their terminal chains. Overexpression of Lewis antigens, namely, sialyl Lewis x and associated antigens, has been seen in pancreatic ductal adenocarcinoma (PDAC) cell lines and tissues. These findings point to the possibility that tumor indicators, glycoproteins containing sLex, are released into the bloodstream by pancreatic tumors [9]. Hematogenous metastasis is hypothesized to be strongly related to the determinants sLea and sLex, which are involved in glycan-mediated adherence of cancer cells to vascular endothelium. Therapeutic applications for blocking this cell adhesion pathway may inhibit hematogenous metastasis [10]. In vivo evidence supports the hypothesis that when sLe-x/a structures are overproduced in cancer cells, these facilitate the bonding of circulating cancer cells with selectins of endothelial cells, hence facilitating the emergence of metastases. E- and P-selectins were also shown to have a substantial function in the peritoneal dissemination of pancreatic cancer cells [11].

## Review

Methodology

Systematic Literature Search 

This study was conducted to ascertain the functionality of CA 19-9 in pancreatic tumor identification and progression. Preferred Reporting Items for Systematic Reviews and Meta-Analyses (PRISMA) guidelines, 2020, were followed in this study [12]. An exhaustive search of the published literature from 2013 to 2023 was carried out using databases (Google Scholar, Cochrane Library, PubMed Central, or PMC, and PubMed). Articles were considered without restrictions of place, time, age, or gender. Grey literature, editorials, conferences, books, and documents were excluded. The investigation proceeded using key terms like "CA 19- 9" or "carbohydrate antigen 19- 9, "pancreatic neoplasms," "pancreatic cancer," "early diagnosis," "sensitivity and specificity," "biomarkers," "tumor markers, biological," "diagnostic accuracy," "diagnostic performance," "prognostic value" "early detection" "serum marker" and "tumor antigen," which were combined using Booleans. For the PubMed database, the MeSH approach was used. Table 1 comprises information on databases and the search strategy.

**Table 1:** Databases used for collecting articles (along with search strategies and appropriate filters) PMC: PubMed Central, CA 19-9: carbohydrate antigen 19-9

Databases searched	Keywords used and search strategy created	Filters applied	Articles identified
PubMed	"CA 19-9" AND "pancreatic cancer" AND "early diagnosis” AND Early pancreatic cancer, pancreatic tumors, pancreatic cancer recurrence, pancreatic neoplasms, prognostic factors for pancreatic cancer, and pancreatic cancer detection OR pancreatic cancer diagnosis OR ( "Pancreatic Neoplasms/blood"[Majr] OR "Pancreatic Neoplasms/chemically induced"[Majr] OR "Pancreatic Neoplasms/complications"[Majr] OR "Pancreatic Neoplasms/diagnosis" [Majr] OR "Pancreatic Neoplasms/immunology"[Majr] OR "Pancreatic Neoplasms/mortality" [Majr] OR "Pancreatic Neoplasms/prevention and control"[Majr] ) AND CA 19-9	Free full text, meta-analysis, narrative reviews, systematic reviews, randomized trials over the last 10 years (2013-2023), human studies, and articles in the English language.	95
PMC	("ca-19-9 antigen"[Supplementary Concept] OR "ca-19-9 antigen"[All Fields] OR "ca 19 9"[All Fields] OR "ca-19-9 antigen"[MeSH Terms] OR ("ca-19-9"[All Fields] AND "antigen"[All Fields])) AND ("pancreatic carcinoma"[All Fields] OR "pancreatic cancer"[All Fields] OR "pancreatic neoplasms"[MeSH Terms] OR ("pancreatic"[All Fields] AND "neoplasms"[All Fields]) OR "pancreatic neoplasms"[All Fields] OR ("pancreatic"[All Fields] AND "cancer"[All Fields])) AND ("early diagnosis"[MeSH Terms] OR ("early"[All Fields] AND "diagnosis"[All Fields]) OR "early diagnosis"[All Fields]) AND ("diagnosis"[Subheading] OR "diagnosis"[All Fields] OR "screening"[All Fields] OR "mass screening"[MeSH Terms] OR ("mass"[All Fields] AND "screening"[All Fields]) OR "mass screening"[All Fields] OR "early detection of cancer"[MeSH Terms] OR ("early"[All Fields] AND "detection"[All Fields] AND "cancer"[All Fields]) OR "early detection of cancer"[All Fields])	Published during the last 10 years (2013-2023)	50
Cochrane Library	"CA 19-9" AND "pancreatic cancer"	Published during the last 10 years (2013-2023)	7
Google Scholar	“Pancreatic cancer” AND “CA 19-9” AND “tumor markers”	Published during the last 10 years (2013-2023)	74

Article Extraction

All the articles from different databases were collected and noted after the removal of duplicates. These identified articles were screened based on the title first and later with abstract screening along with satisfying eligibility criteria. This was followed by skimming full-text articles for relevance. The finalized papers were checked for quality and potential bias.

Inclusion Criteria 

We incorporated specific criteria for inclusion. Only papers composed entirely in English and freely accessible in a full-text format, within the last decade (2013-2023), were considered. All the original articles, observational studies, and review articles (narrative reviews, systematic reviews, meta-analysis), and valid study designs were included in this review. Both randomized controlled trials (RCTs) and those without randomization were considered. Also, there were no restrictions regarding place, time, age, or gender.

Exclusion Criteria

The current systematic review did not incorporate any investigation into animal studies. Studies without sufficient data or results were excluded. Grey literature, editorials, conferences, commentaries, guidelines, books, and documents were also excluded.

Quality Assessment

The task was to evaluate the quality and potential for bias in the studies incorporated into the analysis. We employed suitable lists, such as the Scale for the Quality Assessment of Narrative Review Articles (SANRA), AMSTAR-2 (a measurement tool to assess systematic reviews), Newcastle-Ottawa scale, Joanna Briggs Institute (JBI) quality assessment checklist, and Cochrane risk of bias tool, to assess the methodological soundness of the study, encompassing aspects such as study design, patient recruitment, measurement of the index test (CA 19-9), reference standard, and credible sources of bias. It is advisable to consider the quality assessment outcomes when interpreting the findings.

Results

**Figure 1:**
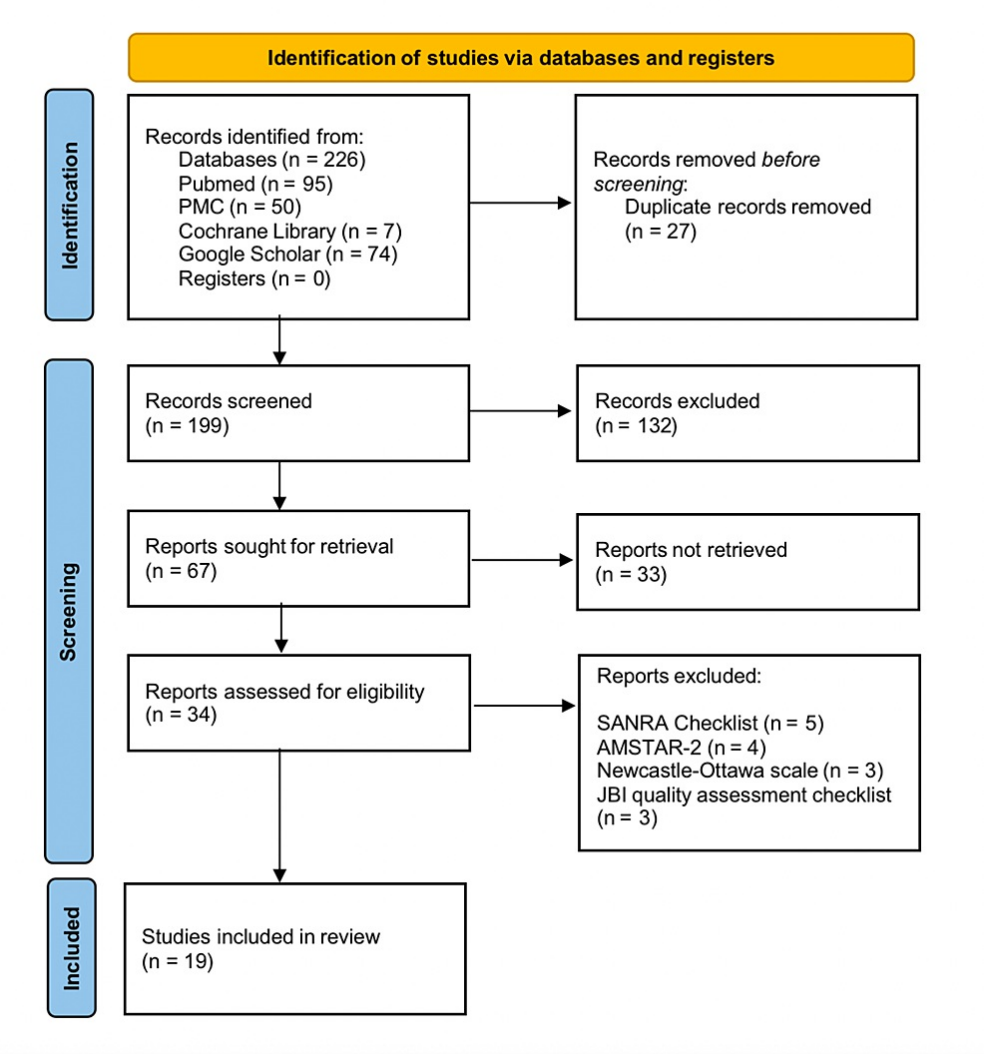
PRISMA flowchart for the current systematic review n: number, PRISMA: Preferred Reporting Items for Systematic Reviews and Meta-Analyses, SANRA: Scale for the Quality Assessment of Narrative Review Articles, AMSTAR-2: Assessment of Multiple Systematic Reviews 2, JBI Critical Appraisal Checklist: Joanna Briggs Institute critical appraisal checklist

A corpus of 226 articles covering the last 10 years (January 2013 to May 2023) was gathered from the databases mentioned above using search techniques and pertinent filters. The screened articles underwent quality assessment after duplicate and unnecessary records were removed. Meta-analyses and systematic reviews were assessed with the help of the AMSTAR-2 software; narrative reviews were evaluated using the SANRA checklist. The JBI quality assessment checklist was used to assess case series and case reports, while RCTs were assessed using the Cochrane risk assessment checklist. Non-RCTs and observational studies were evaluated using the Newcastle-Ottawa checklist. The PRISMA chart, as shown in Figure 1, provides a comprehensive rundown of the screening and article selection process. Table 2 includes extensive information on the quality assessment tools used for the systematic review.

**Table 2:** Description of quality assessment tools used to access the studies included RCT: randomized controlled trial, SANRA: Scale for the Quality Assessment of Narrative Review Articles, AMSTAR-2: Assessment of Multiple Systematic Reviews 2

Quality assessment tools used	Article type	Maximum score	Score required to be included	No. of included studies
SANRA checklist	Narrative/traditional review	12	>9	7
Newcastle-Ottawa scale	Non-RCT and observational studies	9	6-8	7
AMSTAR-2	Systematic review and meta-analysis	16	12-15	4
Cochrane risk of bias assessment tool	RCT	7	5	1

Discussion

CA 19-9: Sensitivity and Specificity

The study by Giannis et al. showed that among suspected individuals with symptoms, carbohydrate antigen 19-9 had a median accuracy of 79% to detect cancer (sensitivity), whereas the ability to be elevated, particularly in PC (specificity), was 82%. They also emphasized, based on recent research, that new biomarkers are constantly being added to the toolkit for PDAC screening, prediction, and prognosis [13]. Daamen et al. performed a systematic review to determine the significance of CA 19-9 in detecting PC recurrence post-surgery. A thorough search was done up till October 2, 2017. The diagnostic utility of different tumor markers was evaluated. The results from four different studies were used to draw forest plots. Pooled values showed that the biological marker had a specificity of 0.83 as well as a sensitivity of 0.73 that was statistically significant in identifying recurrence [14]. A study by Chang and Kundranda mentioned that although the CA 19-9 marker is commonly employed for PDAC detection, it is not specific. According to the study, the average accuracy to detect pancreatic cancer (sensitivity) was 80% in people with symptoms, and the extent of the marker to remain specific only to PDAC (specificity) was 82%-90%. The ability of the marker to show elevation in PDAC patients (positive predictive value, or PPV) and the power of the marker to remain normal in non-PDAC patients (negative predictive value, or NPV) were 72% and 81%-96%, respectively. The wide variation in specificity was noted and was primarily attributed to the rise of CA 19-9 in many other conditions (tumorigenic as well as non-tumorigenic/inflammatory) apart from PDAC [15].

Swords et al. in their study included a report by Steinberg, from 1990, that summarized a mean sensitivity as well as specificity of 81% and 90%, respectively, based on 24 studies with a cohort of 1040 patients and 3282 controls. This article used an upper range of 37-40 U/mL to derive these values. Since then, numerous pieces of published research have referred to these sensitivity and specificity (S and S) estimations [16]. An observational study conducted by Parikh et al. determined that a CA 19-9 cut-off value of 88 U/mL is optimal for distinguishing PDAC from other differentials, using receiver operating curve (ROC) analysis. The improved specificity with the higher-than-average cut-off of CA 19-9 was evidenced at 80% from 66% with the average cut-off, but the sensitivity dropped from 85% to 66% [17]. An updated meta-analysis was carried out by Poruk et al. in which people who had previously been given a diagnosis of a non-cancerous pancreatic condition were used as the control group. A cohort of 3285 people with pancreatic cancer identified from 57 studies was included in the analysis. They had specificity estimates encompassing 1882 patients with benign illnesses affecting the pancreas. Overall, the calculated specificity was around 82.80%, while the calculated sensitivity was 78.20%. The 95% confidence (CI) was 79.9%-85.3% and 76.1%-80.2%, respectively; the sensitivity estimate matched what Steinberg reported [18]. Table 3 includes conclusions of the studies included for sensitivity as well as specificity.

**Table 3:** Studies included for CA 19-9 sensitivity and specificity PDAC: pancreatic ductal adenocarcinoma, CA 19-9: carbohydrate antigen 19-9, PPV: positive predictive value, NPV: negative predictive value

Serial no.	Author	Year	Type of study	Outcome
1	Giannis et al. [13]	2021	Narrative review	This study aimed to systematically investigate, evaluate, and report an overview of PDAC; values of 79% and 82% for sensitivity as well as specificity of CA 19-9 in symptomatic individuals, respectively, were reported.
2	Daamen et al. [14]	2018	Systematic review	In monitoring pancreatic cancer recurrence, this research investigated serum markers. The conclusions of different studies were included, and then forest plots were created. The statistically significant sensitivity and specificity were 0.73 and 0.83, respectively.
3	Chang and Kundranda [15]	2017	Narrative review	Pancreatic cancer marker CA 19-9's sensitivity and specificity in symptomatic individuals were 79%-81%, with a specificity of 82%-90%. This range was because of the elevation of CA 19-9 levels in other cancerous and non-cancerous hepatopancreatic biliary conditions.
4	Swords et al. [16]	2016	Narrative review	The average specificity, as well as sensitivity, were 81% and 90%, respectively, when the higher limit was fixed at 37-40 U/mL.
5	Parikh et al. [17]	2015	Observational study	The specificity, sensitivity, PPV, and NPV of 66%, 85%, 83%, and 69%, respectively, were determined using a standard CA 19-9 threshold value at 37 U/mL. The sensitivity, specificity, PPV, and NPV were 66%, 80%, 72%, and 75%, respectively, when the CA 19-9 cut-off was increased to 88 U/mL, hoping for better differentiation from its differentials, proving the increase in specificity at a higher cut-off.
6	Poruk et al. [18]	2013	Systematic review and meta-analysis	By including patients with known benign pancreatic illnesses as controls, whether CA 19-9 is specific for PDAC and how sensitive the population is, was re-evaluated by experts. The mean specificity was shown to be 82.8% (95% CI 79.9%-85.3%), while the mean sensitivity was discovered to be 78.2% (95% CI 76.1%-80.2%).

CA 19-9 for Pancreatic Adenocarcinoma Screening

In their 1998 study, Zhao et al. presented findings indicating that the sensitivity for PDAC diagnosis ranged from 25% to 80.0% when utilizing a single marker out of the 13 known tumor markers of PDAC. However, when employing two markers, the sensitivity increased to a range of 75%-96.77%. Remarkably, the sensitivity reached 100% when utilizing more than three markers. These results initiated the thought that simultaneous assessment of markers while screening on a vast scale may help boost detection rates of pancreatic cancer [19]. Based on this study, Yang et al. conducted simultaneous measurements of more commonly used biomarkers like serum CA 19-9, pancreatic elastase-1, pancreatic lipase, and pancreatic amylase. The objective was to assess the precision of individual markers and combinations of two, three, or four markers for early detection of PDAC. Surprisingly, including lipase, amylase, or elastase-1 in the diagnostic process did not yield enhanced diagnostic accuracy, and the detection rate fell to 17.1% when all four abnormal markers were used [19].

In an assessment of 16 screening studies in people with known PDAC risk factors, the early identification increased the cure rate following resection to 60% from 25% (p=0.011). It improved the survival of patients to 14.5 months from four months (p=0.001). CA 19-9 has been widely investigated and considered an ultimate PDAC biomarker until Hanna-Sawires and colleagues gave a different viewpoint. They included a meta-analysis of CA 19-9's diagnostic utility in which a total of 13 studies were included; a specificity between 68% and 80% and a sensitivity of 80% were reported concluding that CA 19-9's diagnostic precision is unsatisfactory even in those at considerable risk of PDAC [20]. A worse prognosis is the fate of advanced cancer when CA 19-9 levels are high. A spike in CA 19-9 has been linked to cancers of the pancreas, but it may also be caused by a wide variety of benign inflammatory illnesses of the colon, rectum, stomach, and uterus. In addition, certain people with a particular Lewis genotype do not express CA 19-9; only 65% of patients at the resectable stage had abnormally high marker levels. It is not advised to use this biological parameter as a PDAC screening marker because of the aforementioned factors, according to the comprehensive investigative studies selected by Hasan et al. [21].

According to a meta-analysis included by Loosen et al., the positive predictive value of CA 19-9 was low (0.5%-0.9%). It had a poor sensitivity (75.5%) and specificity (77.6%) for the diagnosis of PDAC. Hence, it is not a reliable screening measure. In addition, the statistical data indicate that around 5%-10% of individuals of Caucasian descent exhibit the Lewis null phenotype; it can be inferred that the diagnostic efficacy of CA 19-9 is further diminished [22]. In a comprehensive screening conducted by Kim et al., a total of 70,940 asymptomatic participants were screened; among them, a cohort with elevated CA 19-9 levels was investigated, and an insignificant number of individuals (four) were identified with pancreatic cancer. The study demonstrated a detection sensitivity reaching 100% and a specificity of around 98.50%. However, the PPV was found to be only 0.9% [15,16]. In a distinct investigation conducted in Japan, a cohort of 13,000 unaffected people was examined, resulting in identifying merely four cases of PC and establishing a PPV of 0.03%. An inadequate PPV narrows the usefulness of CA 19-9 as a screening measure [15]. Table 4 includes the inferences of the studies that included CA 19-9 as a marker for pancreatic adenocarcinoma screening.

**Table 4:** Studies with CA 19-9 for pancreatic adenocarcinoma screening PDAC: pancreatic ductal adenocarcinoma, CA 19-9: carbohydrate antigen 19-9, PPV: positive predictive value

Serial no.	Author	Year	Type of study	Outcome
1	Yang et al. [19]	2022	Observational study	Inclusion of lipase, amylase, or elastase-1 along with CA 19-9 in the diagnostic process did not yield early diagnostic accuracy, and the detection rate fell when all four abnormal markers were used, suggesting PDAC can be accurately determined using CA 19-9 alone.
2	Hanna-Sawires et al. [20]	2021	Narrative review	Early identification and resection of PDAC increased the cure rate and survival rate of patients, but the accuracy of CA 19-9 was insufficient for the use of screening and early detection.
3	Hasan et al. [21]	2018	Narrative review	Elevated CA 19-9 levels indicated advanced PDAC. However, variable specificity and unexpressivity in the Lewis null population made CA 19-9 an unreliable PDAC screening marker.
4	Loosen et al. [22]	2016	Narrative review	CA 19-9 had a negligible PPV (0.5%-0.9%) and poor sensitivity (75.5%) and specificity (77.6%) for the diagnosis of PDAC. Hence, it was not recommended as a screening tool.
5	Chang and Kundranda [15]	2015	Narrative review	A large screening study conducted by Kim et al., with a total of 70,940 asymptomatic participants, and an investigation conducted in Japan, with a cohort of 13,000 unaffected people, using CA 19-9 showed a PPV of 0.9% and 0.03%, respectively, demonstrating its constraints as the screening measure attributing to the poor prevalence of PDAC in the general population.

CA 19-9 as a Biological Marker for Early-Stage Pancreatic Carcinoma Detection

​​It was determined via research by Fahrmann et al. that CA 19-9 has an essential benefit of an earlier diagnosis of resectable cancer. In the initial stage, elevated CA 19-9 levels were seen as early as two years before clinical diagnosis, with the sensitivity reaching 50% and specificity reaching 99% within six months before diagnosis. It also proved its accuracy of 64%, 46%, and 30% in distinguishing PC in healthy individuals, pancreatitis, and benign cysts, respectively, at a significant specificity of 99%. Also, when CA 19-9 alone did not succeed in cancer diagnosis, a 13.2% sensitivity spike in pancreatic cancer detection could be achieved when markers like LRG1 and TIMP1 were used together with CA 19-9 [23]. The patients diagnosed with PDAC demonstrated atypical processing of apolipoprotein (APO), producing various isomers. In a prospective research, Honda et al. used the isoform of APO (ApoA2-ATQ/AT) to check its accuracy in detecting pancreatic cancer in its early stages. When compared to CA 19-9, which had a 36% sensitivity to detect cancer within 18 months before the diagnosis of PDAC, the APO isoform, along with CA 19-9, had an improved sensitivity of 43% at 98% specificity. Despite the fact that that sensitivity took a moderate spike even after the combination of CA 19-9 and Apo isomer, a 98% specificity is significant enough to prove the worth of combination markers in the initial stages [24].

Kunovsky et al. backed up these arguments by recommending that future research look into whether combining CA 19-9 with other indicators can increase sensitivity and specificity, increasing its utility in screening and diagnosis [25]. After an in-depth search of articles, finalized studies showed that on integrating measurements of the commonly used CA 19-9 marker assay with the identification of the same antigen marker (CA 19-9) expressed on mucin (MUC5AC and MUC16), the ability to detect PC was enhanced compared to relying solely on the antigen marker and was observed across all sample sets, leading to a sensitivity range of 67%-80% with 98% specificity. Integration with cell migration-inducing hyaluronan-binding protein (CEMIP) levels holds promise as a novel laboratory indicator of pancreatic ductal adenocarcinoma. The utilization of CA 19-9 and CEMIP demonstrated a substantial enhancement in both sensitivities and specificity for distinguishing individuals with PC across all stages, as well as discerning patients with initial (first/second) stage pancreatic cancer from those who are unaffected at all. A novel biomarker, namely, PC-594, which is a metabolite of serum fatty acid, demonstrated enhanced specificity in identifying pancreatic ductal adenocarcinoma compared to the conventional biomarker CA 19-9 [25].

Pancreatic cancer presents similarly to chronic pancreatitis, making suspicion of cancer negligible in the initial stages. Hence, improving the ability to differentiate these is useful in early-stage detection. Up to 40% of chronic pancreatitis patients had increased CA 19-9 levels, indicating that these values cannot distinguish pancreatic cancer from chronic pancreatitis. In earlier meta-analyses, serum CA 19-9 was shown to be very accurate in distinguishing pancreatic cancer from benign pancreatic illnesses, including chronic pancreatitis. In contrast, Su et al. found an overall positive likelihood ratio (PLR) of 4.08, suggesting that pancreatic cancer patients have around four times higher chances of having increased CA 19-9 levels compared to chronic pancreatitis patients and a negative likelihood ratio (NLR) of 0.24, implying that 24% of known PC patients did not have raised antigen marker levels. This rate is too high to completely exclude the possibility of pancreatic cancer in those who test negative for CA 19-9. This makes it hard for CA 19-9 to differentiate malignant and benign cases with CA 19-9 alone. While supporting a cancer diagnosis when combined with other clinical and pathological data, high CA 19-9 levels should indicate pancreatic carcinoma [26]. O'Brien et al. performed a case-control study in which the ELISA and CLIA measurements were used to study CA 19-9 along with other biological markers, carcinoembryonic antigen-related cell adhesion molecule 1 (CEACAM1) and regenerating islet-derived protein 3 alpha (REG3A). These marker tests, along with a fourth marker, CA 125, were done at various stages prior to the diagnosis of PDAC. CA 19-9 (>37 U/mL) was shown to have a sensitivity of 68% until 12 months and 53% until 24 months before diagnosis at 95% improved specificity in this investigation. Since 20% of PDAC patients who tested negative for CA 19-9 were shown to have elevated CA 125 levels, detection of these 20% of cases may be achieved by evaluating CA 125 together with CA 19-9 [27].

Several researchers have sought a CA 19-9 protein biomarker test. Park et al. performed a study with 182 cases stratified as early and advanced stages, with 66 individuals serving as controls stratified as pancreatitis patients and healthy individuals. CA 19-9 alone had a 71% sensitivity [20]. When paired with CA 19-9, high plasma thrombospondin-2 (TSP-2) levels were found by Kim and colleagues to distinguish PDAC patients from healthy controls with 87% sensitivity and 98% specificity. APO-E, A1, and L1 were used as markers by Liu and fellow researchers, in addition to inter-alpha-trypsin inhibitor heavy chain H3 (IATIH3), for improving the diagnostic ability of pancreatic carcinoma. In their study, 80 already diagnosed cancer patients served as cases; 30 and 40 benign cases and healthy individuals, respectively, served as controls. When this new set of markers was used in conjugation with known marker CA 19-9, it improved the sensitivity up to 95% at 94.1% specificity. Despite encouraging outcomes, we need more data to reproduce and confirm these effects in pre-diagnostic settings as these studies had few patients [20]. Similarly, using both CA 19-9 and CA 242 biomarkers yielded a heightened sensitivity of 89%, but without affecting the specificity. However, in contrast to the findings of O'Brien et al., it is essential to note that despite this improvement, neither of these conventional biomarkers (CA 19-9 and CA 242) demonstrated the ability to serve as a specific and dependable means for identifying PC at the initial stage itself [22]. Table 5 includes conclusions of the studies including CA 19-9 as a biological marker for early-stage pancreatic carcinoma detection.

**Table 5:** CA 19-9 as a biological marker for early-stage pancreatic carcinoma detection IATIH3: inter-alpha-trypsin inhibitor heavy chain H3, TSP-2: thrombospondin-2, APO: apolipoprotein, CEMIP: cell migration-inducing hyaluronan-binding protein, CA 19-9: carbohydrate antigen 19-9, CA 242: carbohydrate antigen 242, CA 125: carbohydrate antigen 125, CEACAM1: carcinoembryonic antigen-related cell adhesion molecule 1, REG3A: regenerating islet-derived protein 3 alpha

Serial no.	Author	Year	Type of study	Outcome
1	Fahrmann et al. [23]	2021	RCT	CA 19-9 has an essential benefit of earlier diagnosis of resectable cancer in asymptomatic persons. When CA 19-9 alone did not succeed in cancer diagnosis, a 13.2% sensitivity spike in pancreatic cancer detection coulb be achieved when markers like LRG1 and TIMP1 were used together with CA 19-9.
2	Hanna-Sawires et al. [20]	2021	Narrative review	A study showed that CA 19-9 alone had 71% sensitivity. When examined along with TSP-2, it reached 87% and hit 95% when paired with IATIH3 and a set of apolipoproteins APO E, A1, and L1.
3	Honda et al. [24]	2019	Systematic review and meta-analysis	Combining CA 19-9 with Apo isoforms significantly increased the accuracy of early pancreatic cancer diagnosis compared to using either marker alone.
4	Kunovsky et al. [25]	2017	Narrative review	Pairing CA 19-9 with other indicators like CA 19-9 expressed on mucin (MUC5AC and MUC16) and CEMIP demonstrated a substantial enhancement in both sensitivity and specificity. Therefore, a workup in this direction may improve CA 19-9 utility in PDAC detection.
5	Loosen et al. [22]	2017	Narrative review	Utilization of both CA 19-9 and CA 242 biomarkers yielded a sensitivity of 89%, but without affecting the specificity. None of these serve to be a reliable indicator to detect PDAC at an early stage.
6	Su et al. [26]	2015	Meta-Analysis	Blood CA 19-9 levels alone cannot distinguish pancreatic cancer from chronic pancreatitis, thereby making it unlikely for early pancreatic cancer detection.
7	O'Brien et al. [27]	2014	Observational study	Measuring CA 19-9, CA 125, along with novel markers CEACAM1 and REG3A, showed that CA 19-9 together with CA 125 had an improved sensitivity by detecting 20% of cases that were CA 19-9 negative.

CA 19-9 as a Prognostic Indicator of Pancreatic Tumors

Prognostic data, the ability to stratify patients into various survival groups, can be gleaned from determining the amounts of CA 19-9 in serum. It also helps doctors decide whether or not a pancreatic tumor can be removed surgically. Accurate prognosis determination is crucial to choose the appropriate treatment and to prevent recurrence. It can be achieved by assessing the findings that are associated with developing high-grade tumors. One such association was seen recently between tissue biomarkers that cause alterations at gene levels and PDAC. To determine the significance of this association, Mehta et al. conducted an observational study where a known marker for PDAC, CA 19-9, was evaluated along with another group of markers like S100A2, S100A4, and CA 125. This study found that these tumor markers had incredible potential to detect pancreatic carcinoma and strongly showed an inverse association between survival and the presence of three tumor markers (excluding the S100A2 marker). The survival rate declined significantly by 16 months when more than one marker was present. Nevertheless, the researchers also believed that these markers have the ability to detect recurrences in treated patients [28]. The previously reported range of CA 19-9 levels, indicative of resectability, is 92.8-622 U/mL. The prognosis was assessed over a broad span of CA 19-9 levels in a population research by Mirkin et al., which included 4701 patients. No association was found between CA 19-9 ≤800 and survival benefit among individuals who received the primary surgical intervention (p>0.05, for all stages I, II, and III). Moreover, significantly worse survival was shown at every stage of the disease tested when levels were over 800 (p≤0.0001). Neoadjuvant treatment (NAT) recipients showed levels >800 were not related to advanced illness (p>0.05, in stages II and III). In contrast, a shorter survival advantage was seen in early disease (p<0.05, in stage I). Mortality risk was 3.29 times higher for those with levels >800 than those with levels at 100 in the multivariate analysis (p<0.0001) [29].

The prognosis of PDAC patients was shown to be highly linked with aberrant blood CA 19-9 and carcinoembryonic antigen (CEA) levels. Wu et al. concluded that patients with above-normal CA 19-9 levels alone or along with above-normal CEA levels often had a worse prognosis compared to typical values. It may be because rising tumor markers levels, such as CA 19-9 and CEA, are indicative of a more advanced stage of tumor development. Independent prognostic variables in PDAC patients were shown to include metastatic potential through nodal spread, CA 19-9, and CEA level above their respective cut-offs, as determined by Cox logistic analysis of the results. Even though the research found a reduced positive incidence of CEA in patients included, a rise in CEA is often associated with a worse prognosis [30]. Likewise, a retrospective study by Distler et al. demonstrated that preoperative levels of CEA and CA 19-9 in patients with PC correlated with survival benefits following resection. The median survival time for first-group patients having low pre-surgery CEA levels together with low pre-surgery CA 19-9 values was 33.3 months (95% CI 25.1-41.5). The second group consisted of patients with either above-normal levels of CA 19-9 or above-normal levels of CEA. The statistically significant survival advantage for this group was 28.5 months. Group 3 patients with both markers elevated had a mean survival of 23.9 months (95% CI 13.9-33.9). In cases where resectability or operability is questionable, it is vital to evaluate blood levels of CEA and CA 19-9 before surgery [31].

Poruk et al. gathered different studies that compared serum CA 19-9 levels less than 37 U/mL and less than 120 U/mL ahead of surgical resection in PDAC-diagnosed patients with patients having above-normal CA 19-9 levels of more than 400 U/mL. The results showed that survival among patients with low levels was markedly improved post-treatment. A statistically significant survival benefit of additional seven months was seen among patients with regular antigen marker levels compared to patients with above-normal levels. Chemotherapy-based treatment almost doubled the survival when the below-normal levels compared to levels more than 1167 U/mL, hence, proving the significance of CA 19-9 in predicting progression after treatment [18]. Table 6 includes outcomes of the studies that included CA 19-9 as a prognostic indicator of pancreatic tumors.

**Table 6:** CA 19-9 as a prognostic indicator in pancreatic tumors CA 125: carbohydrate antigen 125, CA 19-9: carbohydrate antigen 19-9, CEA: carcinoembryonic antigen, PDAC: pancreatic ductal adenocarcinoma

Serial no.	Author	Year	Type of study	Outcome
1	Mehta et al. [28]	2022	Observational study	S100A2 and S100A4 markers were evaluated along with CA 125 and CA 19-9 markers. It showed a negative association between the overall survival and the presence of three biomarkers other than S100A2. The survival rate declined significantly by 16 months when more than one marker was present.
2	Mirkin et al. [29]	2021	Observational study	No association was found between survival benefits and antigen biomarker levels below 800. However, pre-treatment CA 19-9 levels >800 were correlated inversely with long-term survival and may be indicative of severe illness.
3	Wu et al. [30]	2021	Observational study	Preoperative blood levels of CA 19-9 and CEA were strongly related to PDAC patient's survival rates and may be utilized to assess prognosis.
4	Poruk et al. [18]	2017	Systematic review and meta-analysis	When CA 19-9 levels were not markedly elevated, surgical resection or chemotherapy improved survival. PDAC prognosis was independently predicted by pre-treatment CA 19-9 levels.
5	Distler et al. [31]	2013	Observational study	Preoperative CEA levels and CA 19-9 levels were inversely associated with patient survival advantage after resection.

Limitations

The current review mainly incorporated studies from January 2013, which may have missed previous studies. Excluding studies that were not free and published in languages other than English might have excluded a few high-valued articles. Also, any gender- or age-specificity was not considered. The present report limited its focus to one potential tumor marker, lacking studies on other possible markers. The variability observed in the studies incorporated in the analysis regarding their research methodology, demographic, characteristics of the participants, and the criteria used for diagnosing CA 19-9 could have impacted the outcomes. Furthermore, the studies exhibited varying levels of quality, and publication bias may have been included in the article.

## Conclusions

This study aimed to detect the potential of carbohydrate antigen 19-9 as a biological parameter for detecting pancreatic carcinoma at an early stage. The review found that although this marker was found to have similar sensitivity and specificity to detect pancreatic adenocarcinoma in symptomatic patients and recurrent cases, its PPV was significantly low, making it unreliable for screening in the general population when used alone. However, CA 19-9 showed moderately improved diagnostic accuracy and effectiveness when conjugated with other markers as well as when used in high-risk cohorts. The data provided evidence that CA 19-9 might be helpful as a biomarker for detecting pancreatic cancer in its early stages. However, extensive research into existing or new biomarkers that can synergistically improve outcomes must be employed. The included studies concluded that CA 19-9 is helpful as a prognostic marker and can be used when resectability or operability is in doubt. It can also be used to predict the course of disease following treatment.

Subsequent research endeavors ought to employ uniform procedures, augment the sample size, and account for subgroup analyses predicated on tumor stage, tumor location, and other pertinent factors. Nevertheless, integrating additional diagnostic techniques and clinical assessment along with CA 19-9 is crucial to detect pancreatic carcinoma before it reaches an incurable stage. Consequently, advances in discovering novel diagnostic features become imperative. Combining existing markers and new potential markers may prove valuable in further therapeutic approaches, therefore shedding light on the disease's fundamental underpinnings. Future strategies for pancreatic cancer are likely to incorporate molecular techniques, such as miRNA analysis and characterization, as part of the investigative advances.
